# Exploring confidence in recognizing oral cancer among dentists and dental students

**DOI:** 10.1007/s00784-025-06675-w

**Published:** 2025-12-04

**Authors:** Kamran Ali, Daniel Zahra, Sadeq Ali Al-Maweri, Mahwish Raja

**Affiliations:** 1 Qatar University, QU Health College of Dental Medicine, Doha, 2713 Qatar; 2 Peninsula Medical School, Faculty of Health, University of Plymouth, Plymouth, PL4 8AA UK

**Keywords:** Cognitive bias, Dentists, Dental students, Diagnosis, Oral cancer

## Abstract

**Objectives:**

To explore correlation between ability of dentists and dental students to recognise the clinical presentation of oral cancer and their self-rated confidence.

**Methods:**

A cross-sectional, analytical design was used for this study. A validated oral disease recognition scale (ODRS) encompassing a range of benign, premalignant, and malignant oral conditions was used for data collection. For each of the seven patient cases, the participants were asked to provide a diagnosis and rate their self-perceived confidence. Confidence ratings were used as a predictor in a logistic regression model to determine its value as a predictor of diagnosis accuracy, controlling for other factors.

**Results:**

A total of 252 participants completed the online survey. All correlations between pairs of Cases are positive and statistically significant (*p* < 0.001). An ordinal logistic regression model predicting Correct vs. Incorrect based on Confidence, Case, participant status, participant gender, and participant age group (and a random effect for participant) suggests that each unit increase in confidence results in a 0.235 unit increase in log-odds of a correct response (an Odds Ratio of 1.265, *p* < 0.001).

**Conclusions:**

Overall, confidence ratings appear to predict diagnosis accuracy, after controlling for case and participant characteristics. Notwithstanding the limitations of the current study, the oral disease recognition scale may serve as a useful tool to assess clinicians’ ability to recognize oral cancer and identify potential cognitive biases in their clinical assessment skills.

**Clinical relevance:**

This study highlights the correlations between diagnostic accuracy and confidence of dentists and dental students in identifying oral cancer, which is crucial for timely referral and improved patient outcomes. By identifying gaps in diagnostic confidence and potential cognitive biases, the findings can inform targeted educational interventions for reliable oral cancer screening in general dental practice.

## Introduction

Regular dental visits serve as a crucial platform for the opportunistic screening of oral cancer, making dental professionals the frontline defenders in early detection. All dental professionals including general dentists, specialists, dental hygienists, and dental therapists have an ethical and professional duty to perform thorough clinical oral examinations during patient assessments. This includes systematically inspecting and palpating oral tissues to identify suspicious lesions—such as persistent ulcers, erythroplakia, leukoplakia, or unusual growths that may indicate potentially malignant disorders (PMDs) or oral cancer [1, 2]. Given that early recognition and urgent referral to specialists significantly influence treatment outcomes, dental teams play a pivotal role in improving survival rates through timely intervention [3].

Beyond detection, dental professionals must also deliver preventive counselling, educating patients on modifiable risk factors like tobacco use, alcohol consumption, and HPV infection, while promoting healthy lifestyle choices [4–9]. By integrating routine screenings, risk assessment, and patient education into standard practice, the dental community can substantially reduce the burden of advanced oral cancer and enhance overall public health outcomes [10].

Dental professionals need to balance prompt recognition of suspicious lesions with the discernment to identify benign conditions that do not require urgent specialist intervention. Inappropriate referral of innocuous lesions under the urgent suspected cancer pathway can overwhelm specialist services, delaying care for high-risk patients who truly need immediate attention [11–13]. Dental professionals can refine their diagnostic acumen through continuous education and evidence-based guidelines to distinguish between benign mucosal changes and clinically significant abnormalities, such as oral leukoplakia, erythroplakia, and oral squamous cell carcinoma. This approach ensures efficient resource allocation, prioritizes urgent cases, and maintains the efficiency of referral pathways [11, 12].

Published literature has highlighted gaps in the abilities of dental professionals to recognize a wide variety of soft and hard tissue pathologies with confidence. Cognitive bias resulting from overconfidence or under confidence can adversely impact on clinical decision-making and patient outcomes [14–16]. While numerous studies evaluated dental professionals’ self-reported knowledge and practices on oral mucosal lesions diagnosis and oral cancer screening, only limited studies assessed the clinical skills of dental professionals’ in recognizing oral mucosal lesions [17]. Hence, he aim of this study was to explore correlations between ability of dentists and dental students to recognise the clinical presentation of oral disease and their self-rated confidence. To the best of authors’ knowledge, this is a maiden study to explore overconfidence bias amongst dental students and dentists in regard to clinical detection of oral cancer.

## Materials and methods

### Research ethics approval

Ethical approval for this study was provided by the Institutional Review Board (IRB), X University (Approval Number: *QU-IRB 1849-EA/23)* This study was conducted in compliance with the ethical principles outlined in the declaration of Helsinki for research involving humans, including research on identifiable human material and data. Participation in the study was voluntary and all data were collected and processed anonymously. All participating students gave informed consent before responding to the survey.

### Study design and settings

A cross-sectional analytical design was employed for this research. The study was conducted from 30th June to 30th October 2024.

### Sampling technique and recruitment of participants

A purposive non-randomized sampling technique was used to target dentists as well as dental students. Dental students and qualified dentists working as clinical supervisors were recruited from a dental school The dental students were in Years 4 and 5 of the dental programme and were undertaking clinical training. The participants were invited to participate by approaching the head of the institution. The administrative office forwarded the invitation to eligible participants using their professional email. The invitations were accompanied by a participant information sheet explaining the purpose and scope of the study and that participation entailed completing an online questionnaire. Reminders were sent two weeks and four weeks after the initial invitation.

### Sample size calculation

The required sample size was calculated using power analysis with G*Power software (version 3.1) [18]. The required sample size was estimated to be between 255 with 8–12 degrees of freedom, α = 0.05, and a power of 0.90 to detect small-to-medium effects (w = 0.2). These parameters were also appropriate to detect small effect sizes in mean scores between independent groups.

### Data collection instrument

A previously validated scale, namely, The Oral Disease Recognition Scale (ODRS) was used for data collection [19]. The scale included seven patient cases encompassing a range of benign, premalignant, and malignant oral conditions as listed below:

#### Case 1

Hairy tongue.

#### Case 2

Leukoplakia.

#### Case 3

Pyogenic granuloma.

#### Case 4

Lichen planus.

#### Case 5

Oral squamous cell carcinoma.

#### Case 6

Herpes zoster.

#### Case 7

Facial nerve palsy.

Each case included a brief case history and a clinical image depicting key clinical signs. Five possible diagnoses were provided for each case. The participants were required to provide the most appropriate diagnosis for each case and to rate their confidence on a scale ranging from 1 (least confident) to 10 (extremely confident) for the diagnosis of each case.

### Data collection

The questionnaire for the study was distributed electronically via Google Forms. The initial section asked participants to provide informed consent, confirming that their participation was voluntary. The second section collected demographic details, including age, gender, institution, and professional status. The third section involved seven patient cases, each accompanied by a case history and clinical image. Participants were asked to select the most appropriate diagnosis from five options provided for each case.

### Data analysis

Data were analyzed using the R statistical environment for Windows (R Core Team, 2022) and SPSS version 28.0 (IBM Corp. 2021 Armonk, NY: USA). Descriptive statistics were calculated for each case, and confidence plotted against accuracy for each case by subgroups. Confidence ratings were used as a predictor in a logistic regression model to determine its value as a predictor of diagnosis accuracy, controlling for demographic factors including age, gender, and professional status of the participants.

## Results

### Descriptives

A total of 252 participants completed the survey. Of those, age group data was missing for 1 (0.40%), and Gender data for 2 (0.79%); these were replaced with ‘Missing’ but otherwise included in the analyses. Professional status was completed by all respondents (dentist/dental student). Table 1 provides a summary of the demographic characteristics of the participants. The sample was relatively balanced in regard to gender, with a slight majority of female participants (53.17%). The study population was predominantly young, with the largest group being 18–25 years old (56.75%). This indicates that most participants were in the early stages of their dental careers. Reflecting the age data, the vast majority of participants were students (71.83%), while a smaller proportion (28.17%) were qualified professionals (dentists).

**Table 1:** Summary of demographic characteristics

Factor	Subgroups	*n*	% Total
Gender	Female	134	53.17
	Male	116	46.03
	Missing	2	0.79
Age	18–25	143	56.75
	26–35	63	25.00
	Over 35	45	17.86
	Missing	1	0.40
Status	Professional	71	28.17
	Student	181	71.83

### Participant confidence and diagnostic accuracy by case

Table 2 shows a summary of overall confidence ratings and diagnostic accuracy by case. Participants’ confidence and diagnostic accuracy was highest for case 1 and lowest for case 7. The findings show that confidence is not a reliable indicator of competency in diagnosis for certain types of lesions (especially case 7 and, to a lesser extent, case 4). Targeted training is needed to address specific cognitive errors, particularly for the clinical presentation mimicked by case 7, where a high rate of error coincides with unjustified confidence.

**Table 2:** Overall *c*onfidence *r*atings by *c*ase

Case	Min	Max	Mean	SD	Item-Total^1^	Accuracy^2^
1	1	10	7.21	2.73	0.70	69.84
2	1	10	6.56	2.65	0.74	60.32
3	1	10	6.96	2.90	0.70	63.10
4	1	10	6.90	2.73	0.71	55.16
5	1	10	6.66	2.72	0.69	62.70
6	1	10	6.06	2.75	0.77	53.57
7	1	10	6.33	2.76	0.74	34.92

^1^ Item-Total correlation, with item excluded from total and only complete cases used

^2^ Percentage of participants who answered correctly

### Correlation between confidence ratings for each case

All correlations between pairs of cases are positive and statistically significant (all *p* < 0.001) as summarized in Table 3. These findings suggest, while individual participants may have rated their confidence higher or lower on specific cases compared to others, overall confidence ratings across the seven cases were positively correlated.

**Table 3:** Pearson correlation coefficients for relationship between confidence ratings on each case

	Case 2	Case 3	Case 4	Case 5	Case 6	Case 7
**Case** **1**	0.645	0.536	0.585	0.477	0.615	0.550
**Case** **2**		0.613	0.624	0.508	0.577	0.603
**Case** **3**			0.618	0.551	0.559	0.509
**Case** **4**				0.530	0.542	0.530
**Case** **5**					0.654	0.637
**Case** **6**						0.728

### Comparison of confidence and accuracy by group

**Fig. 1:**
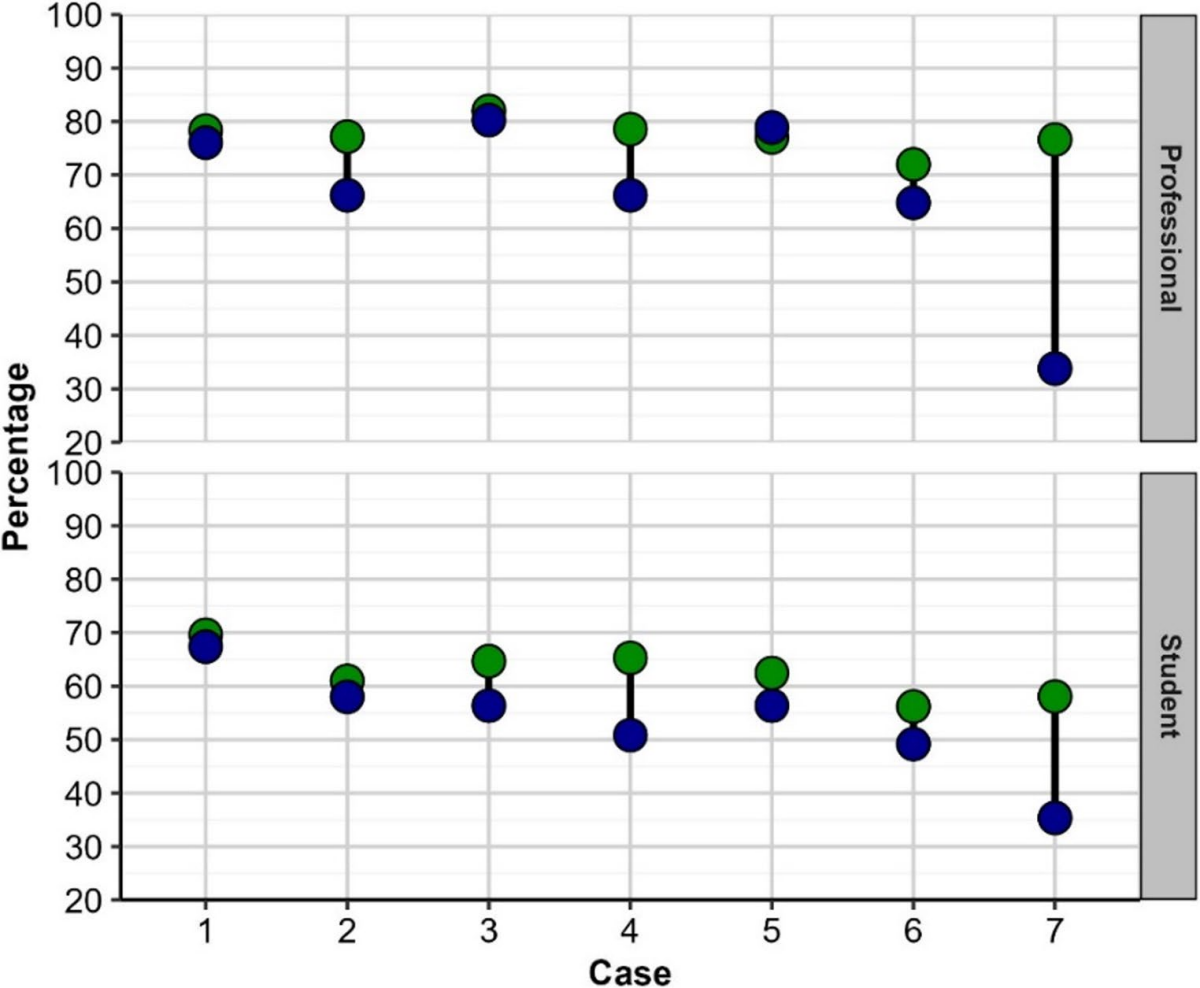
Accuracy (blue circles) and confidence ratings (green circles), converted to percentages, for each case by status group

Confidence ratings (converted from a 1–10 rating scale to %) and accuracy (facility for each case; proportion of group answering correctly) for each case by group membership; professionals (dentists) compared to students are depicted in Fig. 1. Green points represent confidence ratings; blue pints represent accuracy; length of the connecting lines represents difference. The results presented in Fig. 1 showed that the confidence ratings of dentists were consistently higher compared to dentists across all seven cases. The gaps between dentists and dental students were widest for more difficult cases (Case 7, followed by case 4 and 6). This trend follows a predictable pattern and underscores that clinical experience after graduation may enhance confidence of dental clinicians.

**Fig. 2:**
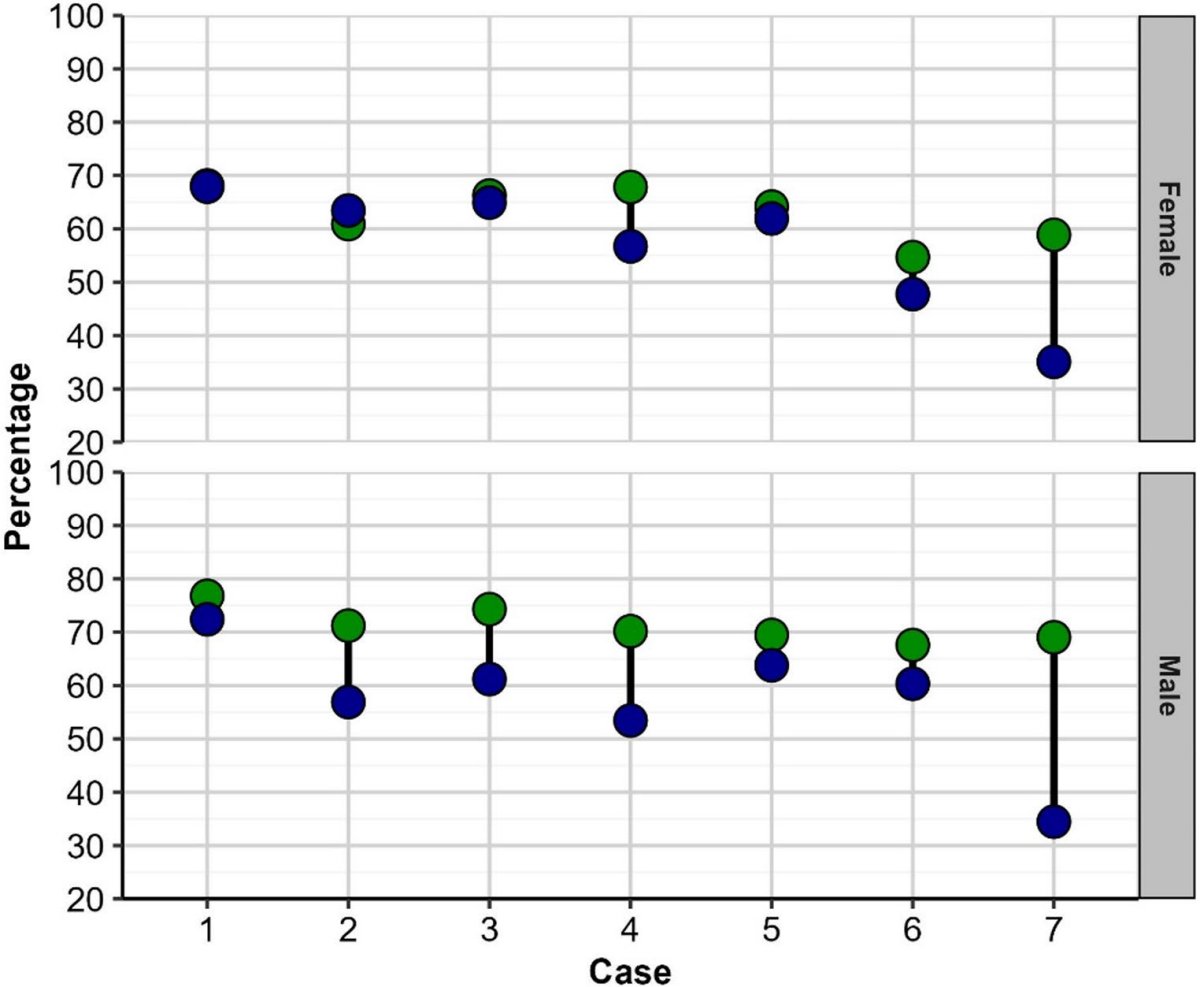
Accuracy (blue circles) and confidence ratings (green circles), converted to percentages, for each case by gender group (female vs. male)

Correlations between confidence ratings and accuracy of diagnosis for each case by gender (males compared to females) are illustrated in Fig. 2. Female participants demonstrated higher diagnostic accuracy than male clinicians across all seven clinical cases. The difference in performance is particularly pronounced in the most challenging cases. Case 7 was the most difficult case for all participants (as previously noted, with only 34.92% overall accuracy). However, female clinicians significantly outperformed their male counterparts. This pattern was also observed for case 4 and case 6 also show very large gaps, indicating that the more challenging the diagnosis, the greater the advantage held by female clinicians in this study. For less difficult cases, such as case 1 (the easiest, with 69.84% overall accuracy) and case 5, the performance gap was considerably narrow. This implies that for straightforward, classic presentations of oral cancer, the diagnostic accuracy between genders is much more similar.

**Fig. 3:**
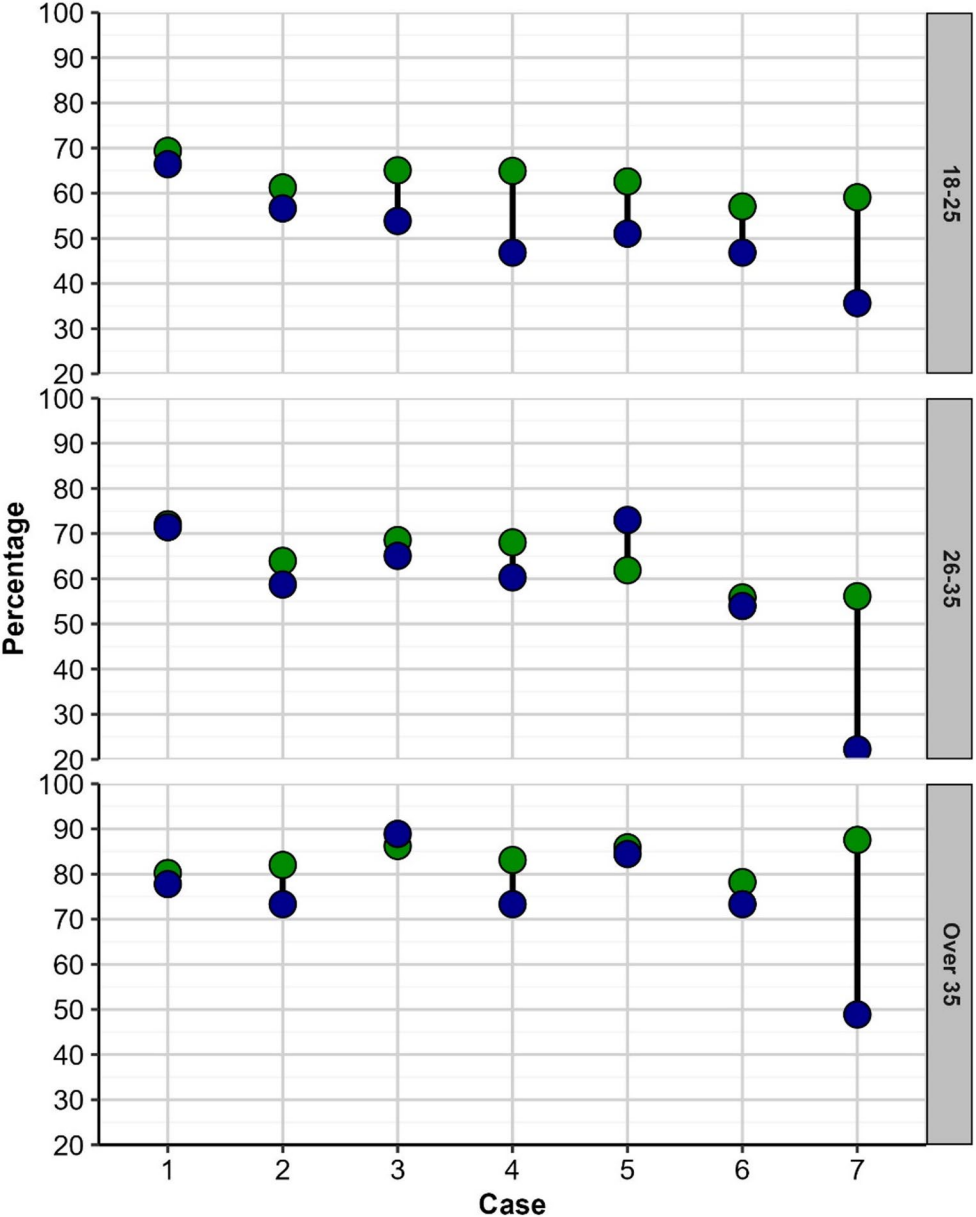
Accuracy (blue circles) and confidence ratings (green circles), converted to percentages, for each case by age-group (18–25 vs. 26–35, vs. Over 35)

Correlations between confidence ratings and accuracy of diagnosis for each Case by age, 18–25-year-olds compared to 26–35-year-olds compared to over 35- year-olds are summarized in Fig. 3. Participants in the older age group (Over 35) demonstrated higher diagnostic accuracy than younger participants (Under 35) across all seven clinical cases. The gaps was more pronounced in case 7 (most difficult case) and narrower in case 1 (easiest case). This pattern aligns well with established theories of expertise and clinical skill development.

An ordinal logistic regression model predicting correct vs. incorrect based on confidence, case, participant status, participant gender, and participant age group (and a random effect for participant) suggests that each unit increase in confidence results in a 0.235 unit increase in log-odds of a correct response (odds ratio = 1.265; *p* < 0.001). The confidence ratings appear to predict diagnosis accuracy, after controlling for case and participant characteristics. Across all demographic groups, participants confidence ratings also reflect their accuracy in diagnosing those cases, with the possible exception of case 7, where all groups show a larger discrepancy between confidence ratings and diagnostic accuracy. Overall, the results showed a satisfactory correlation between confidence ratings and diagnosis accuracy of the participants. Nevertheless, good predictive value between confidence and diagnostic accuracy does not completely rule out all types of cognitive biases and the results should be interpreted accordingly.

## Discussion

The current study focussed on core clinical assessment skills expected from dentists and senior dental students involved in providing clinical care to patients. The oral disease recognition scale is a validated scale based on a range of benign, premalignant, and malignant conditions affecting the orofacial region and may be used for evaluation of clinical oral examination skills. All dental professionals including general dentists, specialists, dental hygienists, and dental therapists should be competent to carry out a comprehensive soft tissue examination for oral cancer screening. Early detection of potentially malignant disorders (PMDs) and oral cancers is critical for improving patient outcomes, and all members of the dental team play a vital role in initial recognition and referral [20, 21]. Dental visits provide an opportunity for oral cancer screening and given the rising global incidence of oral cancer, competency in early detection of oral cancer is important for all dental professionals involved in providing clinical services [22–25]. The current study explores the competence of dentists and dental students to recognize oral cancer on clinical inspection. A systematic review on cost effectiveness of oral cancer screening also reported that visual examination remains the most cost-effective method in this regard [23]. Evidence from the literature suggests that regular dental visits may allow early recognition of oral cancer and facilitate a timely referral [24, 25]. Gaps in the knowledge and oral examination skills of dental students and dentists to recognize oral cancer are widely reported in the literature and broadly corroborate with the results of the current study [26–32]. and the current study aligns Although the participants in the current study showed a low overconfidence bias, the findings do not entirely rule out other cognitive biases, such as confirmation bias (favoring information that supports preexisting beliefs) or anchoring (relying too heavily on initial information) [14–16, 33].

It is noteworthy that discrepancies were observed for participants’ responses to case 7 as the predictive value of confidence and diagnostic accuracy was lower. Given the study employed a quantitative design, the data collected were limited to measurable variables such as diagnostic accuracy and confidence ratings. Such data do not capture the underlying cognitive processes or contextual factors that might explain *why* a particular case was more difficult to diagnose. Case 7 was based on an adult patient with facial nerve paralysis (Bell’s palsy) involving the left side of the face. Bell’s palsy is a type of lower motor neuron paralysis which leads to a full-face paralysis on the ipsilateral side [34]. The corner of the mouth on the affected side is pulled toward the unaffected, or normal, side and results in a characteristic facial asymmetry. However, to a casual observer, this presentation may be mistaken for the involved side, especially if they have a superficial understanding of the pathogenesis of facial nerve paralysis [35]. Although most participants were able to identify facial nerve paralysis, some participants opted for the wrong side. This may be due to a lack of understanding of applied anatomy and pathogenesis of Bell’s palsy, or intuitive thinking rather than an analytical approach may explain the discrepancies in diagnostic accuracy and self-perceived confidence ratings. Nevertheless, it reflects a real-world scenario and may prompt dental professionals to recognize how time constraints in clinical practice may impact on their diagnostic skills.

The knowledge and skills to detect oral cancer accurately and with confidence can be enhanced through targeted continuing professional development (CPD) and shadowing specialists in maxillofacial surgery oral medicine specialists. Regular training updates ensure dental professionals remain adept at recognizing subtle clinical features of suspicious lesions while minimizing unnecessary referrals. Additionally, the growing integration of artificial intelligence (AI) applications in dentistry presents a promising adjunct to clinical judgment [36–38]. AI-driven tools, including image analysis software and risk assessment algorithms, can enhance diagnostic accuracy by flagging high-risk lesions and providing decision-support [39]. It is worth reiterating that that AI-based tools for oral cancer detection should complement, not replace, clinical expertise, emphasizing the need for balanced adoption alongside ongoing professional education.

### Limitations

The main limitations of the study are related to a limited sample size and sampling technique. Although the sample size determined by power analysis was achieved, the findings could have been strengthened by an increased sample size. Moreover, the eligible participants who were invited were constrained by the research team’s professional network which may have introduced potential selection bias, limiting the generalizability of the findings. Therefore, the results may not accurately reflect the broader population of dentists and dental students. Future studies should employ stratified sampling techniques to ensure a more representative sample by accounting for key variables such as professional status, and geographic location to enhance the validity and applicability of findings.

## Conclusion

Notwithstanding the limitations of this study, the findings show that overall, confidence ratings of the dentists and dental students appear to predict diagnosis accuracy, after controlling for case and participant characteristics. The results also show that oral disease recognition scale may serve as a useful tool to assess clinicians’ ability to recognize oral cancer and identify potential cognitive biases in their clinical assessment skills.

## Data Availability

The data underlying this article will be shared on reasonable request to the corresponding author.
